# A Novel Encoder-Decoder Model for Multivariate Time Series Forecasting

**DOI:** 10.1155/2022/5596676

**Published:** 2022-04-14

**Authors:** Huihui Zhang, Shicheng Li, Yu Chen, Jiangyan Dai, Yugen Yi

**Affiliations:** ^1^School of Computer Science and Technology, Qilu University of Technology (Shandong Academy of Sciences), Jinan, China; ^2^School of Computer Engineering, Weifang University, Weifang, China; ^3^School of Software, Jiangxi Normal University, Nanchang, China

## Abstract

The time series is a kind of complex structure data, which contains some special characteristics such as high dimension, dynamic, and high noise. Moreover, multivariate time series (MTS) has become a crucial study in data mining. The MTS utilizes the historical data to forecast its variation trend and has turned into one of the hotspots. In the era of rapid information development and big data, accurate prediction of MTS has attracted much attention. In this paper, a novel deep learning architecture based on the encoder-decoder framework is proposed for MTS forecasting. In this architecture, firstly, the gated recurrent unit (GRU) is taken as the main unit structure of both the procedures in encoding and decoding to extract the useful successive feature information. Then, different from the existing models, the attention mechanism (AM) is introduced to exploit the importance of different historical data for reconstruction at the decoding stage. Meanwhile, feature reuse is realized by skip connections based on the residual network for alleviating the influence of previous features on data reconstruction. Finally, in order to enhance the performance and the discriminative ability of the new MTS, the convolutional structure and fully connected module are established. Furthermore, to better validate the effectiveness of MTS forecasting, extensive experiments are executed on two different types of MTS such as stock data and shared bicycle data, respectively. The experimental results adequately demonstrate the effectiveness and the feasibility of the proposed method.

## 1. Introduction

Time series is the sequence of arranged numbers according to the occurrence time, which is also called dynamic series. The time span can be years, quarters, months, hours, or other factors [1]. In recent years, time series are widely applied in various fields, such as economics, medicine, transportation, and environmental science, which has been attracted much attention [2]. According to the number of observed variables, time series data can be divided into univariate time series data and multivariate time series data [2]. Therefore, how to mine useful information from these time series data becomes a very important task in data mining, machine learning, artificial intelligence, and other fields [3]. As a key and crucial branch of time series data analysis, time series prediction aims to accurately predict or estimate the future events by exploring the past and current data of the single variable or several correlated variables [4]. The former is called univariate time series forecasting; the latter is called multivariate time series forecasting. For example, economists utilized the historical data of stock prices to forecast stock prices or trends [5], medical scientists made use of the biological time data to predict diseases [6], transportation departments explored the historical data of traffic flow to predict congestion [7], and environmentalists employed atmospheric timing data to estimate environmental climate changes [8], etc. Nevertheless, time series data not only contains abundant information but also appears to some complex characteristics such as high dimension, nonlinear, fluctuation, and spatiotemporal dependence, which make accurate time series data prediction become a challenging study hotspot [9].

In the past few decades, time series data prediction has been widely concerned and many methods have been proposed [10]. For instance, traditional statistics-based methods focused on relevant domain knowledge, while learning-based methods are introduced to learn temporal dynamics in a pure data-driven strategy. As a popular learning-based method, deep learning can learn the deep latent features from the input data comprehensively and has become a cutting-edge approach [11].

The traditional statistics-based methods include autoregressive (AR) [12], autoregressive moving average (ARMA) [13], autoregressive integrated moving average, and exponential smoothing models (ARIMA) [14]. Although the above methods can utilize statistical inference to describe and evaluate the relationship between variables, they assumed that the input data has a linear relationship between model structure and the constant variance [15]. Therefore, there are some limitations to dealing with complex time series data containing nonlinear and nonstationary structures, so they cannot effectively obtain accurate predictions.

In order to solve the shortcomings mentioned above, many learning-based methods including support vector machine (SVM) [16], genetic algorithm (GA) [17], AdaBoost [18], and artificial neural network (ANN) [19], which can simulate the complex structures of time series data, have been widely applied to time series prediction task. For example, Dong et al. [16] discussed utilizing SVM for predicting building energy consumption in tropical regions, and they considered that it was superior to other neural networks from the views of performance and parameter selection. Yadav et al. [17] proposed a neuron model based on polynomial structure and used the Internet traffic and financial time series data to conduct forecast experiments, which showed that the neural network (NN) model not only achieved better performance but also greatly reduced the computational complexity and running time comparing with the existing multilayer neural networks. However, building an effective learning-based model needs a large amount of professional data, and the training process requires a high level of computer hardware equipment. Therefore, the application of traditional machine learning models is largely limited.

In recent years, with the improvement of data acquisition and computing power, a novel learning-based method called deep learning has attracted much attention. Deep learning [20] can obtain a higher-level representation of the original input via designing simple and nonlinear modules, which was conducive to learning the feature representation. Convolutional neural network (CNN) [21], recurrent neural network (RNN) [22], and variant models have been successfully applied to time series prediction. Zhang et al. [23] proposed a deep spatiotemporal residual network model to predict the flow of people throughout the city. Jagannatha and Yu [24] developed a bidirectional recurrent neural network (BRNN) for medical events detection in electronic medical records. Nevertheless, RNN and BRNN are easy to suffer from the gradient vanishing and gradient exploding problems. To overcome the drawbacks, the long short-term memory network (LSTM) [25] and the gated recurrent unit (GRU) [26] were developed. Since both LSTM and GRU can keep the historical information for a longer time step, they are widely used in time series data analysis, prediction, and classification tasks. Compared with LSTM, the GRU has a simpler structure and fewer parameters, which can reduce the overfitting risk. For example, Shu et al. [27] presented a new neural network model based on improved GRU to predict short-term traffic flow.

As an unsupervised method, Autoencoder (AE) is also widely applied to feature representation learning [28]. In order to extract better features, the RNN is frequently combined with AE. Xu and Yoneda [29] first used a stacked autoencoder (SAE) to encode the key evolution patterns of urban weather systems and then adopted the LSTM network to predict the PM2.5 time series of multiple locations in the city. Zhang et al. [30] proposed an encoder-decoder model for real-time air pollutant prediction, in which LSTM was the main network. The experimental results indicated that the model can fully extract the data correlations and obtain higher prediction accuracy. In addition, the attention mechanism (AM) [31] has attracted extensive attention in time series data analysis and prediction. Han et al. [32] combined LSTM with AM to predict time series, in which the AM can capture time correlation by calculating weights between nodes and neighboring nodes so that it achieved better performance and provided enlightenment for multivariate time series prediction simultaneously.

Although abundant methods have been developed, their performances are limited since the high nonlinearity and nonstationarity of multivariate time series (MTS) data. To improve the prediction performance, a novel encoder-encoder prediction model is presented, and the contributions are as follows:

1. The proposed model can sufficiently extract significant temporal features of MTS data.
2. As a unit structure, the GRU is adopted to describe sequential characteristics which can reduce model parameters in the procedures of encoding and decoding.
3. The AM is introduced into the decoding process for preferably acquiring the reconstructed MTS data.
4. To strengthen the prediction performance, 1D-convolution operation and AM are further performed based on the reconstructed new MTS data, which possess discriminant and significant characteristics.

The outline of this paper is as follows.  Section 2 reviews the related works, and time series data preprocessing is introduced in Section 3.  Section 4 describes the proposed network structure in detail.  Section 5 illustrates extensive experiments to verify the effectiveness and feasibility of the proposed model.  Section 6 provides some conclusions and future works.

## 2. Related Works

Recently, researchers have proposed extensive time series (TS) and multivariate time series (MTS) prediction methods, which are classified into two categories including machine learning and deep learning methods [9].

### 2.1. Machine Learning Methods

The basic assumption of the statistical methods is that the TS and MTS with simple structures are linearity and stationarity. However, in real applications, the TS and MTS data are collected with complex structures, which have high nonlinearity and nonstationarity and they make the TS and MTS forecasting very difficult. Meanwhile, the machine learning algorithms are usually helpful to improve the prediction accuracy [33], which can analyze the behavior of data over time and are independent of the statistical distribution assumption to extract complex nonlinear patterns.

Specifically, Li et al. [34] firstly proposed a chaotic cloud simulated annealing genetic algorithm (CcatCSAGA), which was used to optimize the robust support vector regression (RSVR) parameters for improving the performance of ship traffic flow prediction. Sahoo et al. [35] designed a novel online multiple kernels regression (OMKR), which successively learned kernel-based regression in an extensible manner. Moreover, its effectiveness was demonstrated on real data regression and time series prediction tasks. Ahmed et al. [33], respectively, adopted multilayer perceptron (MLP), Bayesian neural networks (BNN), radial basis function (RBF), general regression neural network (GRNN), *k*-Nearest neighbors regression (KNNR), classification and regression tree (CART), support vector regression (SVR), and Gaussian process regression (GPR) to perform experiments. This study revealed significant differences between various methods in TS and MTS prediction, and the MLP and GPR methods were the best. Besides, in order to improve the performance, Domingos et al. [36], respectively, combined the ARIMA with MLP and SVR to predict time series. It showed that the hybrid model was better than the single model. Rojas et al. [37] presented a hybrid method integrating an artificial neural network and ARMA model, which achieved outstanding results.

### 2.2. Deep Learning Methods

The deep neural network can surpassingly learn complex data representation [38], which is widely utilized in many tasks, such as image classification, image segmentation, and natural language processing.

A convolutional neural network (CNN) was originally designed to process static image analysis, which can obtain invariant local relations across spatial dimensions [39]. Recently, CNN and its variant methods were also developed for time series data prediction [40], classification [41], anomaly detection [42], clustering [43], and so on. For example, Ding et al. [44] applied the CNN model to stock market prediction. Wang et al. [45] introduced deep learning to develop a probabilistic wind power generation prediction model. In this model, a wavelet transform was used to decompose the raw wind power data into different frequencies. Then, a CNN model was used to learn nonlinear features in each frequency for improving prediction accuracy. Finally, the probability distribution of wind power generation was predicted. Different from the above methods, Oord et al. [46] proposed a new network model called WaveNet, which expanded convolution to improve the long-term dependence requirement of time series. Moreover, the size of the receptive field increased exponentially with the depth of layers. Afterward, Borovykh et al. [47] adopted the WaveNet for multivariate financial time series forecasting.

A recurrent neural network (RNN) is also widely exploited for time series prediction [22]. Since there is a long-term dependence on RNN during the training, it will lead to related gradient explosion and gradient disappearance. Therefore, introducing the gating mechanism into RNN has drawn much attention to overcome these limitations and preserves long-term information of time series data, such as long short-term memory (LSTM) [25] and gated recurrent unit (GRU) [26]. The gated variants of RNN essentially preserve the internal state memory through their recurrent feedback mechanism, which makes them very suitable for modeling the time series data. Moreover, their ability to capture complex nonlinear dependence can be extended from short-term to long-term and cross different variables in multivariate systems. Therefore, the performance of these models is excellent in the time series prediction task. Li et al. [48] built a model combining ARIMA and LSTM to improve the prediction accuracy of high-frequency financial time series. Pan et al. [49] applied the model based on the LSTM network to predict urban traffic flow and greatly improved the prediction effect via the spatial correlation. Filonov et al. [50] proposed a model based on the LSTM network to monitor and detect faults in industrial multivariate time series data. Zhao et al. [51] established a two-layer LSTM model to learn gait patterns presenting in neurodegenerative diseases for diagnostic prediction. Jia et al. [52] developed a spatiotemporal learning framework with a dual memory structure based on LSTM to predict land cover. Huang et al. [53] proposed a sequence-to-sequence framework based on GRU to predict different types of abnormal events. Fu et al. [54] used LSTM and GRU to predict short-term traffic flow, which indicated that the RNN-based methods (such as LSTM and GRU) performed better than ARIMA. Zhang et al. [55] utilized four different neural networks, such as MLP, WNN, LSTM, and GRU, to monitor the small watercourses overflow. Furthermore, the models combining CNN with LSTM or GRU have been frequently applied to time series prediction. Wu et al. [56] explored the GRU network to encode the time mode of each sequence with low-dimensional representation and then combined it with a convolutional network for modeling behavioral time series. Shi et al. [57] presented a ConvLSTM network to predict nearby precipitation which can acquire spatiotemporal correlations well.

Autoencoder (AE) has also been successfully applied in time series prediction and is generally combined with other deep learning methods [58]. Considering the inherent temporal and spatial correlation of traffic flow, Lv et al. [59] used AE as one of the modules to construct a deep learning model. Yang et al. [60] proposed a new host load prediction method, which utilized AE as the precyclic feature layer of the echo state network. Gensler et al. [61] combined AE with LSTM for renewable energy power prediction which was superior to the artificial neural network and physical prediction model. Recently, Prenkaj et al. [62] combined AE and GRU to propose a new strategy for predicting the student dropout e-courses.

## 3. Time Series Data Preprocessing

**Figure 1:**
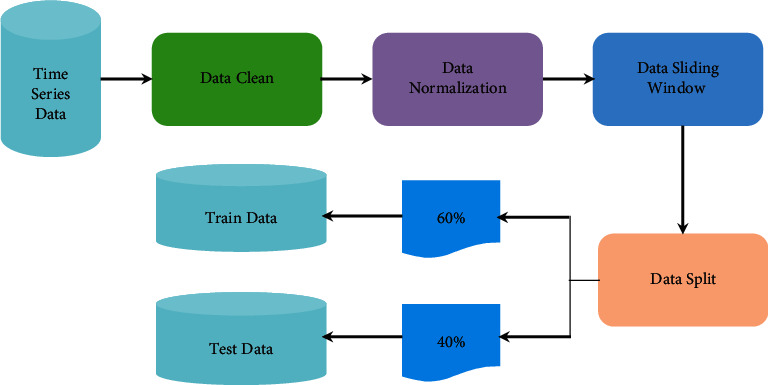
The process of time series data preprocessing.

Generally, time series data are collected manually or automatically; it is difficult to avoid data redundancy, data missing, data error, and other unknown problems in the process of collection and transmission. Therefore, data preprocessing becomes a crucial and necessary procedure for time series data analysis. It mainly includes four stages, such as data clean, data normalization, data sliding window, and data split [63]. The details are illustrated in Figure 1.

**Figure 2:**
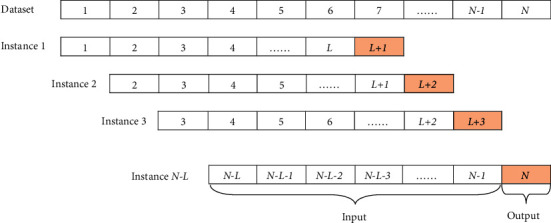
The process of data sliding window for creating a time series data.

(1) *Data Cleaning*. The purpose of data clean is to deal with missing values, outlier values, and redundant attributes in time series data. There are many ways to handle missing and outlier values. One way is to delete the data with missing and outlier values directly. However, when many attributes of data have missing and outlier values, it is very hard to remain adequate useful attributes and results in incomplete time series data, which will affect the learning and generalization ability of models. The other way considers outlier values as missing values and then the data filling technique is applied to solve the above problems. Data filling includes statistics-based and learning-based methods. The former generally adopts mean filling, while the latter adopts simple linear regression or a complex learning model (such as deep learning). In our work, the mean filling is utilized to process missing values and outlier values. Moreover, feature selection or feature extraction methods are generally adopted to solve redundant attributes. In particular, the proposed model in our work is based on a deep learning framework, which has a strong feature representation ability. Therefore, it is robust to deal with data containing redundant attributes.
(2) *Data Normalization*. Since the different attributes of data often have different measurement scales, the values collected may vary widely. For the sake of eliminating the influence of measurement scale and value range among different attributes, it is necessary to perform normalized processing which can scale data in a certain proportion, such as mapping data values to [−1, 1] or [0, 1]. The popular data normalization methods contain minimum-maximum normalization and zero-mean normalization.
Minimum-maximum normalization is named deviation standardization, which maps the values of the original data to [0, 1] via a linear transformation. The formula is as follows:
  (1) $$\begin{array}{c} x^{*}=\frac{x-\min}{\max-\min}, \end{array}$$
where max and min represent the maximum and minimum values of data, respectively. The method can preserve the relationships that exist in original data.
Zero-mean normalization is known as standard deviation standardization. After processing, the mean value and the standard deviation of normalization data are 0 and 1, respectively. The formula is defined as
  (2) $$\begin{array}{c} x^{*}=\frac{x-\bar{x}}{\sigma }, \end{array}$$
where $\bar{x}$ and *σ* are the mean and standard deviation of original data, respectively.
(3) *Data Sliding Window*. This operation mainly creates time series data by the predefined sliding window size and step for the original time series data. In other words, this operation is used to generate the predicted data for the next moment using historical data with a given interval. The specific operation of the data sliding window is shown in Figure 2 [64]. Given any time series data with length *N*, such as {1, 2, 3, 4, 5,…, *N* − 1, *N*}, when the sliding window size is set to *L* and the sliding step is 1, the *N-L* data sets with length *L* + 1 are formed. Particularly, the first *L* data of each set is regarded as training data and the value of the number *L* + 1 is the target value.
(4) *Data Split*. This stage divides the time series dataset into training data and test data. For example, the first 60% are used for training and the remaining 40% are used to test in the experiments.

## 4. The Proposed Method

**Figure 3:**
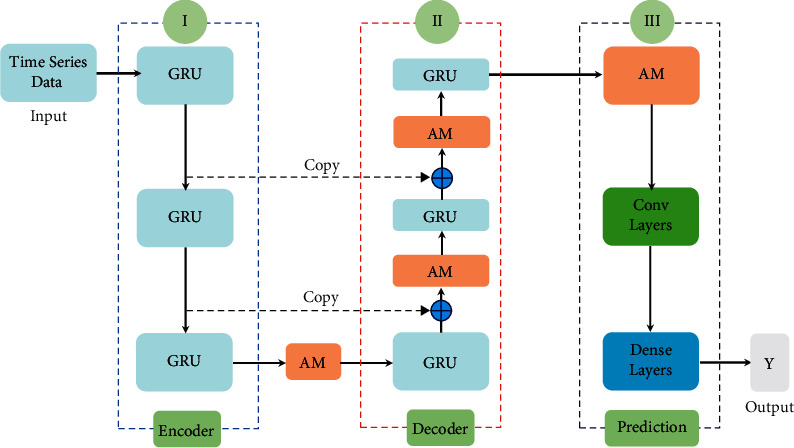
The structure of the proposed network model.

In this work, a novel time series prediction model based on the encoding-decoding framework is designed, which integrates the recurrent neural module, convolutional module, attention mechanism, and fully connection module into a unified framework. As shown in Figure 3, the proposed model consists of three parts such as encoding, decoding, and prediction modules. In the encoding module, the gated recurrent unit (GRU) is taken as the main unit structure for extracting more effective time series features. In the decoding module, the attention mechanism (AM) is introduced to explore the importance of historical data collected at different times, so that it can obtain better new time series data. In addition, taking the influence of previous features on data reconstruction into account, feature reuse is realized by the skip connections based on the residual network. In the prediction module, the convolution layer is adopted to extract effective features from the reconstruction time series. Then, the AM is further performed on the convolution feature mapping owing to the influence of important information on prediction performance. Finally, a multilayer fully connected network is established for prediction.

### 4.1. Deep Autoencoder (DAE)

**Figure 4:**
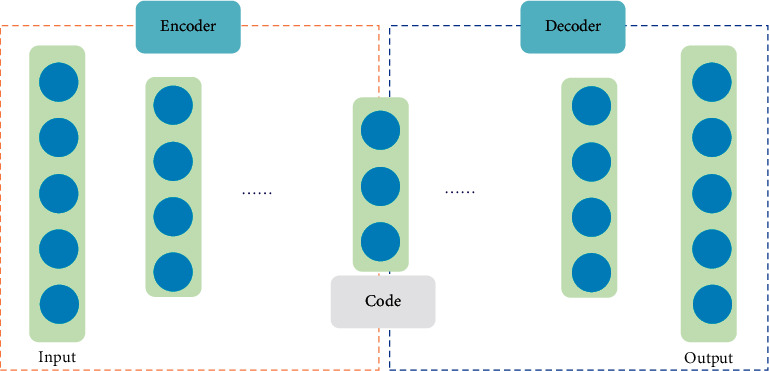
The structure of DAE.

Autoencoder (AE) is an unsupervised deep learning method which is frequently used in feature representation, data compression, image denoising, and other tasks [28]. The structure of AE includes an encoder and a decoder, which only contain a fully connected hidden layer. To better extract features and reconstruct original data, Deep Autoencoder (DAE) [65] is designed that contains multiple hidden layers shown in Figure 4.

### 4.2. LSTM and GRU

In general, DAE is a multilayer feedforward neural network, while it does not consider the importance of historical information of time series data to the prediction or classification of unknown data. As a specific network structure, a recurrent neural network (RNN) [22] can adeptly utilize the historical information of time series data, which adopts a backpropagation through time (BPTT) algorithm to train and learn parameters. However, RNN produced gradient vanishing or gradient exploding problems when it handled time series with long time intervals [25]. In particular, the longer the time interval, the more likely it is to appear severe gradient vanishing or gradient exploding, which will make it difficult to train effective RNN models for long interval sequences.

**Figure 5:**
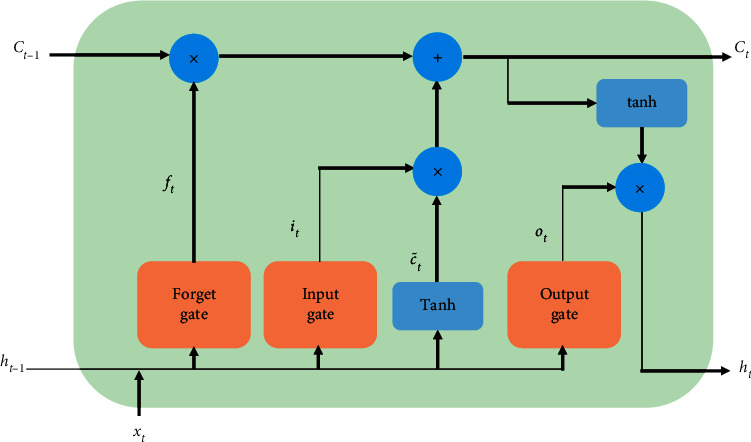
The structure of the LSTM unit.

To solve the above problems, other RNN variants (such as LSTM [25] and GRU [26]) are easier to capture the long-term dependence of time series data. LSTM uses the gate mechanism to control the information accumulation speed and can selectively update information and forget information accumulated. LSTM includes an input gate, forget gate, and output gate, which are displayed in Figure 5. The forget gate *f*_*t*_ controls which information needs to be forgotten derived from the internal state of the previous moment. The input gate *i*_*t*_ controls which information from the current candidate state needs to be retained. And the output gate *o*_*t*_ controls which information of the current internal state needs to be output.

**Figure 6:**
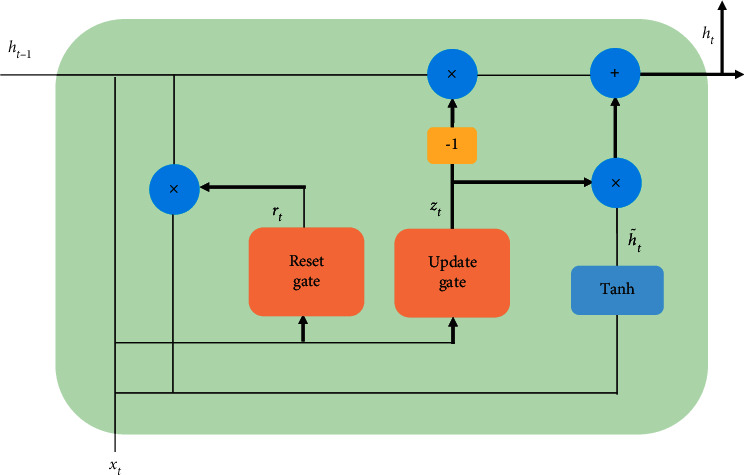
The structure of the GRU unit.

Different from LSTM, GRU is a simplified version of LSTM. It merges the forget gate and input gate into the update gate and retains the original reset gate, as shown in Figure 6. It can be observed that no additional memory units are needed in GRU. It is due to the fact that an update gate can control how much information needs to retain from the historical state and needs to receive from the candidate state for the current state. The calculation formula of GRU is

(3) $$\begin{array}{c} z_{t}=\sigma \left(W_{z}x_{t}+U_{z}h_{t-1}+b_{z}\right),\\ r_{t}=\sigma \left(W_{r}x_{t}+U_{r}h_{t-1}+b_{r}\right),\\ {\tilde{h}}_{t}=\tanh\left(W_{h}x_{t}+r_{t}⊙U_{h}h_{t-1}+b_{h}\right),\\ h_{t}=z_{t}⊙h_{t-1}+{\left(1-z_{t}\right)}_{t}⊙{\tilde{h}}_{t}, \end{array}$$

where *z*_*t*_ and *r*_*t*_ represent update gate and reset gate, respectively. *h*_*t*_ is the state of the current moment *t* and $\tilde{h}$ indicates the candidate state. *σ* is the sigmoid activation function that can convert results to [0, 1]. tanh stands for hyperbolic tangent activation function. The symbol ⊙ is the dot product operation of corresponding elements. *x*_*t*_ represents the input of the neural network at time *t*. *W*_*z*_ , *W*_*r*_, *W*_*h*_ and *U*_*z*_, *U*_*r*_, *U*_*h*_ represent the parameter matrix and recurrent weight of the model. *b*_*z*_, *b*_*r*_, and *b*_*h*_ are the offset vector. Compared with LSTM, GRU has a simple structure and fewer parameters because there are fewer gate structures of GRU. Therefore, GRU not only can reduce the model training time and avoid overfitting problems but also can achieve the same results as LSTM and even better than LSTM. In addition, BiGRU is a variant version of GRU. Although BiGRU has better performance than GRU in some cases, the parameter size of BiGRU is bigger than GRU. In order to overcome the overfitting problem, the GRU is adopted as the main unit structure of the autoencoder.

### 4.3. Attention Mechanism

Attention mechanism (AM) has been widely applied to natural language, computer vision, and other fields [66]. It is a resource allocation scheme that uses limited computing resources to process more important information for the information overload problem. Like artificial neural networks, AM originated from human vision and borrowed from human visual attention mechanisms. The core idea of AM is to select the more critical information and ignore the unimportant or irrelevant information to the current task from a large amount of information [66]. At present, plenty of attention mechanisms have been built to solve related tasks, such as spatial attention, channel attention, and mixed attention mechanisms [67].

**Figure 7:**
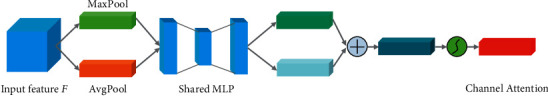
The structure of CA.

In image understanding tasks including image segmentation and target detection, the channel attention (CA) [68] module is mainly adopted to explore relationships between feature maps of different channels, and its structure is shown in Figure 7. In the module, the feature map of each channel is taken as a feature detector that can determine which part of the features should be noticed more. It is well known that the time attribute is very important and also affects the prediction results. Therefore, we view each time attribute as a channel and the channel attention (CA) mechanism is integrated to mine the significance of time attributes in the proposed method.

### 4.4. Prediction Module

**Figure 8:**
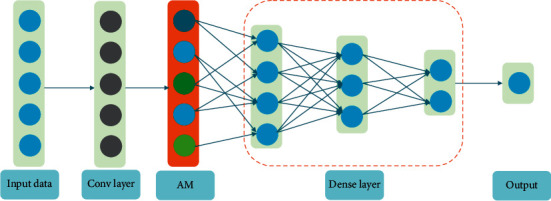
The structure of the prediction module.

In the prediction module, a 1D-convolution is firstly explored to extract features from the time series data reconstructed by DAE. Then, in order to explore the different contributions of historical data for forecasting, the CA mechanism is performed on feature mapping by the previous layer. Finally, a multilayer dense network structure is constructed for prediction. The details are displayed in Figure 8.

## 5. Experiments and Results Analysis

To verify the effectiveness of the proposed method, two series of experiments are conducted on public stock and shared bicycle datasets, respectively, and compared with some related methods. Many experimental results validate the effectiveness of our model.

### 5.1. Evaluation Metrics and Experimental Environment

In order to quantitatively analyze the accuracy and superiority, mean square error (MSE), mean square error (RMSE), mean absolute error (MAE), and mean average percentage error (MAPE) are adopted to evaluate the performance of the proposed model [69]. The calculation formulas are as follows:

(4) $$\begin{array}{c} \text{MSE}=\frac{1}{n}\sum_{t=1}^{n}{\left(X_{t}-X_{t}^{\prime }\right)}^{2},\\ \text{RMSE}=\sqrt{\frac{1}{n}\sum_{t=1}^{n}{\left(X_{t}-X_{t}^{\prime }\right)}^{2}},\\ \text{MAE}=\frac{1}{n}\sum_{t=1}^{n}\left|X_{t}-X_{t}^{\prime }\right|,\\ \text{MAPE}=\frac{100}{n}\sum_{t=1}^{n}\frac{\left|X_{t}-X_{t}^{\prime }\right|}{\left|X_{t}^{\prime }\right|}, \end{array}$$

where *X*_*t*_ and *X*_*t*_′ represent the actual and predicted values of the data and *n* is the number of samples. The smaller the above values, the more accurate the prediction result.

The source codes of the proposed method and the compared methods are completed using Tensorflow with Python. The corresponding versions of the development software and the configurations of the hardware platform are listed in Table 1. Moreover, the settings of the key parameters during the training processing are shown in Table 2.

### 5.2. Stock Data Prediction

#### 5.2.1. Stock Data Description

The stock data used in the experiment are Shanghai Composite Index 50 (SCI-50), CSI-300, and Shenzhen Component Index (SZCI). Each stock data records multiple attributes, such as the closing price, the highest price, the lowest price, the opening price, the previous day's closing price, change, and ups and downs. The closing price, the highest price, the lowest price, and the opening price represent the final price, the highest price, the lowest price, and the first trading price of one stock, respectively. The previous day's closing price is the final price at which a stock is traded on the previous day. Change is the difference between the closing price and the previous day's closing price of the stocks traded (i.e., closing price - previous day's closing price). The value of ups and downs is the change divided by the closing price of the stocks traded (i.e., change/closing price). The details of three stock datasets are listed in Table 3. Meanwhile, Tables 4to 6 give some instances of data and corresponding statistical information for each stock, including the number of records, minimum, maximum, mean, variance, 1/4 value, 1/2 value, and 3/4 value for each attribute. From Tables 3to 5, we can see that there are great differences and fluctuations in the stock data.

#### 5.2.2. Parameters Analysis

Time interval (time step) is the significant factor affecting the prediction of time series data. Therefore, we test the performance of the proposed method with different steps. In the experiment, the time step is set to {5, 10, 15, 20, 25, 30}, and the experimental results are displayed in Tables 7–9. Obviously, in most cases, when the step increases, the value of each evaluation indicator decreases. It indicates that the performance of the proposed model improves with the increasing step. This is because long interval data provides more useful information for prediction. However, as the step continues to increase, the values of each evaluation indicator will increase. It indicates that the performance of the proposed model decreases with the increase of time step. The possible reason is that time series data with too long intervals contains redundant information and high volatility, which makes it difficult to capture more effective information for future data prediction.

#### 5.2.3. Convergence Analysis

**Figure 9:**
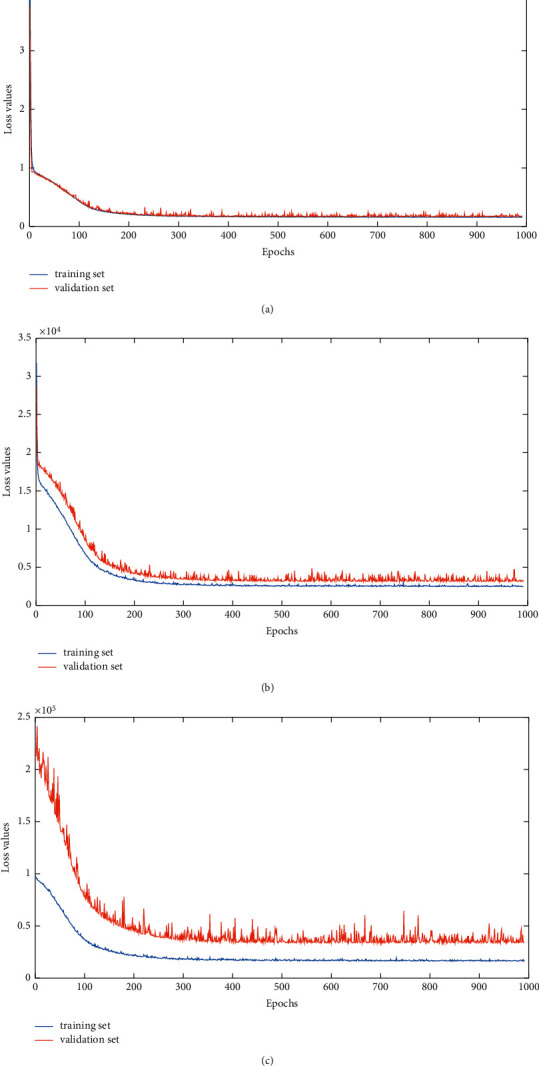
The curves of loss values (MSE) on the training set and validation set of three stock datasets. (a) SCI-50. (b) CSI-300. (c) SZCI.

In order to verify the convergence of our proposed method, we plot the curves of loss values (MSE) on the training set and validation set for each dataset. From Figure 9, we can see that our model reaches convergent very quickly on the training set. For the validation set, the loss values (MSE) of the proposed model fluctuate but basically maintains stability when the number of iteration (Epochs) is greater than 400.

#### 5.2.4. Performance Analysis

In order to further test the performance of the proposed method, we compare it with GRU, BiGRU, GRU-AE, BiGRU-AE, GRU-AE-AM, and BiGRU-AE-AM. Tables 10–12 show the results of different methods on three stock datasets. The following conclusions can be drawn from the experimental results:

1. The performances of traditional GRU and BiGRU models are lower than those of other comparison methods. Furthermore, BiGRU not only makes use of the useful information of historical data in the forward direction but also mines the dependence of current data on historical data in the reverse direction. Therefore, BiGRU has better performance than GRU.
2. The performances of recurrent neural networks (GRU-AE and BiGRU-AE) are superior to the traditional recurrent neural network (GRU and BiGRU). It indicates that introducing encoding-decoding into the recurrent neural network is beneficial to improving the prediction performance of the proposed model.
3. The performances of the recurrent neural network-AE model based on attention mechanisms (GRU-AE-AM and BiGRU-AE-AM) exceed the recurrent neural network-AE model (GRU-AE and BiGRU-AE). It demonstrates that introducing the attention mechanism into the recurrent neural network can mine significant information in time series data.
4. The proposed model is based on the idea of integrating encoding-decoding and attention mechanisms simultaneously into the recurrent neural network. Different from GRU-AE-AM and BiGRU-AE-AM, the proposed method develops the attention mechanism in the decoding stage to capture the degree of importance between different intervals. Therefore, compared with other methods, the presented method establishes significant advantages on different evaluation indicators.

### 5.3. Demand Forecast for Shared Bicycle Data

#### 5.3.1. Shared Bicycle Data Description

The datasets of this experiment are derived from the shared bicycle demand of three streets in Shenzhen, China, such as Longgang Central City, Pingshan Street, and Zhaoshang Street. Each data set contains the historical travel data of shared bicycles, time attribute data (such as hours, working day or not), and weather data (such as temperature, rainfall, wind speed, and humidity). The details are listed in Table 13.

#### 5.3.2. Parameters Analysis

In this experiment, the influence of the time step on the prediction performance is also analyzed adequately. The step size setting is consistent with stock price prediction experiments, and experimental results are shown in Tables 14–16. We can see that the effect of steps differs from the experimental results on the stock data. Firstly, with the increase of steps, the evaluation indicator values of the proposed method decreased on Longgang and Pingshan datasets. However, this trend does not always remain unchanged, and the opposite result will occur when the step continues to increase. Accordingly, the performance of the proposed model will also decrease. Secondly, the results are different from Tables 7–9 and Tables 14–16 on the Longgang Street dataset. When the step is set to the minimum (*L* = 5), the proposed method can obtain the optimal results. The cause is maybe that time series data has strong dependence and complex data structure.

#### 5.3.3. Performance Analysis

Similarly, the proposed method is compared with other well-known methods, and the results are shown in Tables 17–19. On the whole, the experimental results are consistent with those of stock experiments, except for the data in Longgang. In particular, the proposed method can achieve better performance with a step value of 20. It indicates that the data structure is relatively simple, which is prone to overfitting for the complex model. Therefore, the evaluation metrics of the bidirectional recurrent neural network model (BiGRU, BiGRU-AE, and BiGRU-AE-AM) are higher than those of the recurrent neural network model with unidirectional structure (GRU, GRU-AE, and GRU-AE-AM).

## 6. Conclusions and Future Works

In this paper, to improve the accuracy of time series data prediction, the autoencoder, recurrent neural network, attention mechanism, convolution module, and full connection module are integrated to establish a novel prediction model based on an encoding-decoding framework. The prediction performances are evaluated for the stock price and the demand for shared bicycles on three stock datasets and three shared bicycle datasets, respectively. In addition, we compare it with many other related methods, which demonstrate that the proposed model has higher prediction accuracy from the views of multiple quantitative indicators (such as MSE, RMSE, MAE, and MAPE).

The future works mainly include the following points. (1) We will try to apply the proposed model to prediction tasks of time series data in other fields (such as medical, energy, environment, and other industrial data). (2) Using the core idea, we will further extend it to solve the anomaly detection task of time series data. (3) We will intensively study how to combine the traditional multivariate time series method with deep learning to further improve the prediction performance in real applications.

## Figures and Tables

**Table 1:** The description of experimental environment.

*Development software*	*Version*

Python	3.6.0
Tensorflow	2.7.0
System	Window 10 64 bit

*Hardware platform*	*Configurations*

PC machine	Inter core i9 9900k
RAM	32 GB
GPU	GeForce RTX 2080 Ti GPU

**Table 2:** The settings of the key parameters in the training procedure.

Description	Value
Batch-size	256
Optimizer	Adam
Epochs	400
Loss function	MSE

**Table 3:** The details of three stock datasets.

Stock name	Stock code	Start and end time	Number of records
SCI-50	000016	2004.01.02–2021.06.23	4245
CSI-300	399300	2002.01.07–2021.03.17	4657
SZCI	399001	1991.04.04–2021.06.23	7349

**Table 4:** Some data and statistical information of SCI-50.

Data	Closing price	Highest price	Lowest price	Opening price	Previous day's closing price	Change	Ups and downs
2004/1/2	1011.347	1021.568	993.892	996.996	1000	11.347	1.1347
2004/1/5	1060.801	1060.898	1008.279	1008.279	1011.347	49.454	4.8899
**…**	**…**	**…**	**…**	**…**	**…**	**…**	**…**
2012/2/1	1713.684	1751.558	1709.536	1739.638	1744.708	−31.024	−1.7782
2012/2/2	1761.941	1761.941	1714.246	1719.999	1713.684	48.257	2.816
**…**	**…**	**…**	**…**	**…**	**…**	**…**	**…**
2015/2/2	2332.533	2376.426	2329.151	2337.196	2405.38	−72.847	−3.0285
2015/2/3	2405.76	2413.006	2335.107	2362.413	2332.533	73.227	3.1394
**…**	**…**	**…**	**…**	**…**	**…**	**…**	**…**
2021/6/21	3431.252	3455.565	3410.403	3440.744	3454.589	−23.3363	−0.6755
2021/6/22	3464.706	3469.808	3437.955	3444.75	3431.252	33.4535	0.975

Count	4245	4245	4245	4245	4245	4245	4245
Mean	2136.173	2156.355	2113.242	2134.459	2135.591	0.582177	0.043547
Std	811.6843	820.2789	801.5931	811.7199	811.6128	40.36138	1.685313
Min	700.434	706.879	693.528	699.266	700.434	−296.696	−9.4708
25%	1600.299	1614.014	1586.092	1599.408	1599.012	−13.545	−0.7423
50%	2127.203	2150.033	2101.088	2127.804	2127.094	0.493	0.0259
75%	2692.54	2718.884	2666.817	2694.952	2692.181	16.22	0.8297
Max	4731.826	4772.933	4688.263	4726.083	4731.826	296.077	9.6729

**Table 5:** Some data and statistical information on CSI-300.

Data	Closing price	Highest price	Lowest price	Opening price	Previous day's closing price	Change	Ups and downs
2002/1/7	1302.08	1302.08	1302.08	1302.08	1316.46	−14.38	−1.0923
2002/1/8	1292.71	1292.71	1292.71	1292.71	1302.08	−9.37	−0.7196
**…**	…	**…**	**…**	**…**	**…**	**…**	**…**
2009/3/2	2164.666	2177.294	2112.336	2123.367	2140.489	24.177	1.1295
2009/3/3	2142.154	2168.222	2100.644	2109.841	2164.666	−22.512	−1.04
**…**	**…**	**…**	**…**	**…**	**…**	**…**	**…**
2013/1/4	2524.409	2558.529	2498.892	2551.814	2522.952	1.457	0.0577
2013/1/7	2535.985	2545.969	2511.603	2518.047	2524.409	11.576	0.4586
**…**	**…**	**…**	**…**	**…**	**…**	**…**	**…**
2021/3/9	4970.999	5094.311	4917.909	5066.155	5080.025	−109.025	−2.1462
2021/3/10	5003.612	5055.279	4981.616	5047.059	4970.999	32.6127	0.6561

Count	4657	4657	4657	4657	4657	4657	4657
Mean	2762.711	2785.604	2734.602	2760.16	2761.898	0.812626	0.042789
Std	1187.877	1201.201	1171.218	1187.38	1187.571	52.6816	1.65268
Min	818.033	823.86	807.784	816.546	818.033	−391.866	−9.2398
25%	1493.776	1507.972	1472.001	1481.582	1488.291	−16.284	−0.7247
50%	2851.915	2888.093	2818.248	2848.155	2850.829	1.3386	0.069
75%	3607.985	3648.027	3560.634	3605.372	3606.924	20.534	0.8142
Max	5877.202	5930.912	5815.609	5922.071	5877.202	378.179	9.3898

**Table 6:** Some data and statistical information of SZCI.

Data	Closing price	Highest price	Lowest price	Opening price	Previous day's closing price	Change	Ups and downs
1991/4/4	983.11	983.11	983.11	983.11	988.05	−4.94	−0.5
1991/4/5	978.27	978.27	978.27	978.27	983.11	−4.84	−0.4923
**…**	**…**	**…**	**…**	**…**	**…**	**…**	**…**
2010/1/4	13533.54	13782.81	13533.54	13766.1	13699.97	−166.433	−1.2148
2010/1/5	13517.38	13597.36	13324.56	13539.83	13533.54	−16.162	−0.1194
**…**	**…**	**…**	**…**	**…**	**…**	**…**	**…**
2016/1/4	11626.04	12659.41	11625.41	12650.72	12664.89	−1038.85	−8.2026
2016/1/5	11468.06	11687.48	11063.64	11116.9	11626.04	−157.978	−1.3588
**…**	**…**	**…**	**…**	**…**	**…**	**…**	**…**
2021/6/21	14641.29	14721.69	14468.74	14563.05	14583.67	57.6251	0.3951
2021/6/22	14696.29	14706.5	14564.5	14678.37	14641.29	54.9937	0.3756

Count	7349	7349	7349	7349	7349	7349	7349
Mean	6709.184	6778.63	6628.694	6704.283	6707.301	1.885397	0.05939
Std	4325.842	4369.826	4270.334	4322.335	4325.313	153.7217	2.1302
Min	402.5	408.02	397.67	401.57	402.5	−1293.66	−19.7807
25%	3112.336	3134.055	3077.097	3112.637	3111.4	−42.702	−0.8978
50%	4834.614	4867.142	4795.043	4836.637	4831.989	0.381	0.0112
75%	10316.82	10410.65	10223.16	10315	10315.75	51.813	0.9835
Max	19531.16	19600.03	19203.11	19554.58	19531.16	1254.795	26.1963

**Table 7:** The results with different steps on SCI-50.

Time step	MSE	RMSE	MAE	MAPE
5	1682.935	41.024	27.188	**1.023**
10	**1673.594**	**40.910**	**27.266**	1.025
15	1736.988	41.677	27.588	1.036
20	1757.061	41.917	28.304	1.062
25	1752.673	41.865	28.084	1.055
30	1780.636	42.198	28.547	1.072

Bold in the table indicates the optimal results.

**Table 8:** The results with different steps on CSI-300.

Time step	MSE	RMSE	MAE	MAPE
5	3157.709	56.193	36.205	**1.001**
10	**3085.284**	**55.545**	**36.214**	1.005
15	3233.284	56.862	36.904	1.025
20	3287.964	57.341	37.026	1.026
25	3438.429	58.638	39.390	1.082
30	3393.111	58.250	38.940	1.069

Bold in the table indicates the optimal results.

**Table 9:** The results with different steps on SZCI.

Time step	MSE	RMSE	MAE	MAPE
5	34522.267	185.802	127.234	1.186
10	**33851.601**	**183.988**	**127.063**	**1.180**
15	34495.000	185.730	128.299	1.190
20	34767.899	186.462	128.943	1.195
25	36065.960	189.910	132.394	1.226
30	36287.302	190.492	132.812	1.228

Bold in the table indicates the optimal results.

**Table 10:** The results with step value of 10 on SCI-50.

Method	MSE	RMSE	MAE	MAPE
GRU	2356.925	48.548	35.958	1.328
BiGRU	2267.462	47.618	35.466	1.342
GRU-AE	2371.064	48.694	37.074	1.419
BiGRU-AE	1964.477	44.322	31.129	1.164
GRU-AE-AM	2040.477	45.172	31.334	1.164
BiGRU-AE-AM	1814.952	42.602	28.483	1.062
Our method	**1673.594**	**40.910**	**27.266**	**1.025**

Bold in the table indicates the optimal results.

**Table 11:** The results with step value of 10 on CSI-300.

Method	MSE	RMSE	MAE	MAPE
GRU	4262.664	65.289	46.799	1.264
BiGRU	3614.219	60.118	41.625	1.137
GRU-AE	3382.457	58.159	38.588	1.070
BiGRU-AE	3828.393	61.874	44.642	1.244
GRU-AE-AM	3798.575	61.633	41.392	1.127
BiGRU-AE-AM	3726.034	61.041	39.880	1.084
Our method	**3085.284**	**55.545**	**36.214**	**1.005**

Bold in the table indicates the optimal results.

**Table 12:** The results with step value of 10 on SZCI.

Method	MSE	RMSE	MAE	MAPE
GRU	37269.796	193.054	137.383	1.271
BiGRU	35012.771	187.114	126.924	**1.180**
GRU-AE	34737.291	186.379	128.821	1.203
BiGRU-AE	37163.139	192.777	134.793	1.257
GRU-AE-AM	35198.463	187.613	130.611	1.218
BiGRU-AE-AM	34946.619	186.940	131.015	1.216
Our method	**33851.601**	**183.988**	**127.063**	**1.180**

Bold in the table indicates the optimal results.

**Table 13:** The description of shared bicycle datasets.

Dataset	Time	Quantity by hour
Longgang central city	2016.6–2017.8 (except Dec.)	6935
Pingshan street	2016.7–2017.8 (except Dec.)	6935
Zhaoshang street	2016.7–2016.11	2907

**Table 14:** The results with different steps of shared bicycle data on Longgang.

Time step	MSE	RMSE	MAE	MAPE
5	684.764	26.168	17.429	102.576
10	663.629	25.761	16.881	89.556
15	672.780	25.938	16.598	**79.102**
20	**652.445**	**25.543**	**16.421**	88.784
25	726.195	26.948	17.697	93.377
30	695.377	26.370	17.800	123.276

Bold in the table indicates the optimal results.

**Table 15:** The results with different steps of shared bicycle data on Pingshan.

Time step	MSE	RMSE	MAE	MAPE
5	240.870	15.520	11.778	20.356
10	227.618	15.087	11.386	17.991
15	**222.815**	**14.927**	**11.247**	17.931
20	238.981	15.459	12.046	22.808
25	228.705	15.123	11.449	19.670
30	224.910	14.997	11.343	**17.497**

Bold in the table indicates the optimal results.

**Table 16:** The results with different steps of shared bicycle data on Zhaoshang.

Time step	MSE	RMSE	MAE	MAPE
5	**1071.253**	**32.730**	**22.051**	76.338
10	1084.648	32.934	22.286	**58.965**
15	1282.643	35.814	24.423	63.207
20	1201.246	34.659	23.586	63.110
25	1322.340	36.364	24.477	62.043
30	1430.125	37.817	25.936	67.414

Bold in the table indicates the optimal results.

**Table 17:** The results with step 20 of shared bicycle data on Longgang.

Method	MSE	RMSE	MAE	MAPE
GRU	717.634	26.789	17.791	76.668
BiGRU	718.049	26.796	17.711	**63.458**
GRU-AE	784.713	28.013	18.660	106.267
BiGRU-AE	904.714	30.078	22.273	163.759
GRU-AE-AM	740.382	27.210	17.726	85.522
BiGRU-AE-AM	828.928	28.791	18.109	91.725
Our method	**652.445**	**25.543**	**16.421**	88.784

Bold in the table indicates the optimal results.

**Table 18:** The results with step 15 of shared bicycle data on Pingshan.

Method	MSE	RMSE	MAE	MAPE
GRU	308.121	17.553	13.758	21.750
BiGRU	229.627	15.153	11.481	17.345
GRU-AE	275.273	16.591	12.690	19.307
BiGRU-AE	270.095	16.435	12.397	17.419
GRU-AE-AM	212.592	14.581	10.800	15.397
BiGRU-AE-AM	211.903	14.557	10.876	**15.818**
Our method	**222.815**	**14.927**	**11.247**	17.931

Bold in the table indicates the optimal results.

**Table 19:** The results with step 5 of shared bicycle data on Zhaoshang.

Method	MSE	RMSE	MAE	MAPE
GRU	1174.327	34.268	24.864	55.381
BiGRU	1139.840	33.762	24.263	55.748
GRU-AE	1124.142	33.528	24.134	86.665
BiGRU-AE	1180.541	34.359	23.882	72.879
GRU-AE-AM	1241.616	35.237	23.773	46.250
BiGRU-AE-AM	1195.643	34.578	22.705	**43.788**
Our method	**1071.253**	**32.730**	**22.051**	76.338

Bold in the table indicates the optimal results.

## Data Availability

The network code and data are available from the corresponding author upon request.
